# Digenic mutational inheritance of the integrin alpha 7 and the myosin heavy chain 7B genes causes congenital myopathy with left ventricular non-compact cardiomyopathy

**DOI:** 10.1186/1750-1172-8-91

**Published:** 2013-06-21

**Authors:** Teresa Esposito, Simone Sampaolo, Giuseppe Limongelli, Antonio Varone, Daniela Formicola, Daria Diodato, Olimpia Farina, Filomena Napolitano, Giuseppe Pacileo, Fernando Gianfrancesco, Giuseppe Di Iorio

**Affiliations:** 1 Institute of Genetics and Biophysics “Adriano Buzzati-Traverso”, National Research Council of Italy, Naples, Italy; 2 Department of Medical Sciences, Surgery, Neurological, Metabolic and Aging, Second University of Naples, Naples, Italy; 3 Department of Cardiological Sciences, Second University of Naples, Naples, Italy; 4 Department of Neuro-sciences, “Santobono-Pausilipon” Hospital, Naples, Italy

**Keywords:** Left ventricular noncompact cardiomyopathy, Congenital type fiber disproportion, Integrin alpha 7 (ITGA7), Myosin heavy chain 7B (MYH7B), Whole exome sequencing

## Abstract

**Background:**

We report an Italian family in which the proband showed a severe phenotype characterized by the association of congenital fiber type disproportion (CFTD) with a left ventricular non-compaction cardiomyopathy (LVNC). This study was focused on the identification of the responsible gene/s.

**Methods and results:**

Using the whole-exome sequencing approach, we identified the proband homozygous missense mutations in two genes, the myosin heavy chain 7B (*MYH7B*) and the integrin alpha 7 (*ITGA7*). Both genes are expressed in heart and muscle tissues, and both mutations were predicted to be deleterious and were not found in the healthy population.

The R890C mutation in the *MYH7B* gene segregated with the LVNC phenotype in the examined family. It was also found in one unrelated patient affected by LVNC, confirming a causative role in cardiomyopathy.

The E882K mutation in the *ITGA7* gene, a key component of the basal lamina of muscle fibers, was found only in the proband, suggesting a role in CFTD.

**Conclusions:**

This study identifies two novel disease genes. Mutation in *MYH7B* causes a classical LVNC phenotype, whereas mutation in *ITGA7* causes CFTD. Both phenotypes represent alterations of skeletal and cardiac muscle maturation and are usually not severe. The severe phenotype of the proband is most likely due to a synergic effect of these two mutations.

This study provides new insights into the genetics underlying Mendelian traits and demonstrates a role for digenic inheritance in complex phenotypes.

## Background

Congenital fiber type disproportion (CFTD) is a form of congenital myopathy in which consistent type 1 fiber hypotrophy relative to type 2 fibers is the main histological abnormality [1]. The health impairments commonly encountered in CFTD are similar to those of many other congenital myopathies. Muscle weakness is usually relatively stable or slowly progressive during childhood and adolescence. In general, scoliosis and joint contractures (other than mild Achilles tendon contractures) are relatively uncommon. Mutations associated with CFTD have been found in the *TPM3* (MIM# 191030), *ACTA1* (MIM# 102610), *SEPN1* (MIM# 606210), *RYR1* (MIM# 180901), *TPM2* (MIM# 190990) and *MYH7* (MIM# 160760) genes [2-8]. Despite recent advances, no genetic cause has been found in at least 50% of CFTD patients.

Left ventricular non-compaction cardiomyopathy (LVNC) has been reported in patients with different types of neuromuscular disorders but has never been associated with CFTD. Within the last 2 decades, more than 200 cases of LVNC have been described [9]. The hallmark features of this cardiomyopathy include prominent trabeculations and deep endocardial recesses. Symptoms associated with LVNC are variable and can include arrhythmias, thromboembolic events and heart failure. At least 4 genes that cause very rare cardiomyopathy diseases have been found to be linked to non-syndromic LVNC in familial or sporadic cases. Thus far, the X-linked gene known as *G4.5* or taffazin (TAZ) has been found to be associated with the largest number of LVNC cases [10]. Other genes found to be mutated in LVNC include alpha-dystrobrevin (*DTNA*), Cypher/*ZASP* and lamin A/C [11-13]. Cumulatively, these genes have been shown to be associated with only a small percentage of sporadic or familial cases. Over the past few years, mutations in genes encoding sarcomere proteins, the beta-myosin heavy chain (*MYH7*), alpha-cardiac actin (*ACTC1*), cardiac troponin T (*TNNT2*), cardiac myosin- binding protein C (*MYBPC3*), alpha-tropomyosin (*TMP1*) and cardiac troponin I (*TNNI3*) have been identified in a significant proportion of patients with LVNC [14-16].

We report an Italian family in which the proband showed a severe disease phenotype characterized by CFTD and LVNC. The clinical and instrumental analysis of the family members identified the LVNC phenotype in the mother, the sister and the first-degree cousin of the proband, suggesting that the LVNC cardiomyopathy segregated as a dominant mode of inheritance with high phenotypic heterogeneity and reduced penetrance. CFTD phenotype showed a recessive mode of inheritance.

To our knowledge, this is the first example of LVNC cardiac phenotype associated with CFTD. We provide a full description of this new phenotype and of the identification of the disease-causing genes by whole-exome sequencing [17].

## Patients and methods

### Family and unrelated cohort

**Figure 1:**
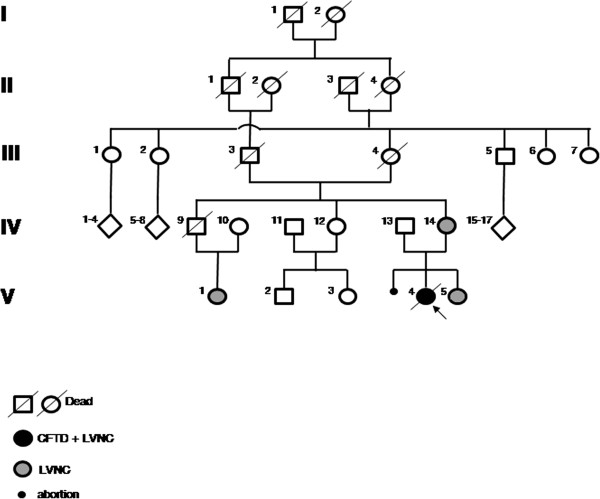
**The complete 5-generational Italian pedigree.** The proband affected by CFTD and LVNC is indicated with an arrow and a dark circle. The mother, sister and cousin of the proband, affected by LVNC, are indicated with gray circles.

A single, multigenerational Italian pedigree was involved in this investigation, as depicted in Figure 1. Phenotypic data were available for 10 individuals (3 males and 7 females) ranging in age from 4 to 50. All individuals were of Caucasian ancestry. DNA was available for 6 individuals (IV-10, IV-13, IV-14, V-1, V-4, V-5). We extended the clinical and instrumental (EMG, ECG, Echocardiography, muscle biopsy) evaluation to the whole family. We identified a mild LVNC phenotype (according to the Jenni criteria) in the mother (IV-14), sister (V-5) and cousin (V-1) of the proband, with hypertabeculations of the mid inferolateral wall and of the ape and without neuromuscular disorders [18]. During the growth, there was a reduction of clinical signs in patients V-1 and V-5. No data have been collected for individual IV-9, who died at the age of 35 of unknown causes. The father (IV-13) of the proband was asymptomatic.

An unrelated cohort (UC) of 24 patients (including LVNC14-BG), all fulfilling the criteria for LVNC, were also analyzed in this study.

Informed consent was obtained from each patient. In the case of minors, parental consent was obtained. The study protocol conforms to the ethical guidelines of the 1975 Declaration of Helsinki, as reflected in the a priori approval by the institution’s human research committee.

### Whole-exome sequencing strategy

For all participants over 18 years of age, DNA was extracted from peripheral blood specimens using a standard salting-out procedure. Genomic DNA samples from two affected females in the pedigree (the proband V-4, affected by CFTD and LVNC, and her cousin V-1, affected by LVNC) were captured with the NimbleGen SeqCap EZ Exome™ capture kits (Roche, Indianapolis, IN, USA)) and sequenced with one lane per sample on an Illumina GAIIx (Illumina, San Diego, CA, USA) with 90-bp paired-end reads.

Sequences were aligned to the human reference genome sequence (GRCh37/hg19) with the MAQ7 and NextGENe software v2.00 with sequence condensation by consolidation (SoftGenetics, State College, PA, USA). This approach resulted in more than 40× of target exome coverage.

Single nucleotide variants (SNVs) were called with MAQ and NextGENe. Small insertions and deletions were detected with NextGENe. Called SNVs were annotated with SeattleSeq Annotation and filtered with dbSNP130.

### Mutation analysis

A mutation analysis of already-known genes for CFTD and LVNC and validation and segregation analyses of the selected variants obtained from exome sequencing, were performed by Sanger sequencing.

Each variant was amplified and sequenced, as described in our previous studies [19,20]. Variations were detected by multiple-sequence alignments using the Autoassembler program (Applied Biosystems).

The SIFT and PolyPhen2 software packages (http://sift.bii.a-star.edu.sg/www/SIFT_seq_submit2.html, http://genetics.bwh.harvard.edu/pph2/) were used to assess the deleterious effects of the mutations.

To assess the minor allele frequencies of the variants, the dbSNP (http://www.ncbi.nlm.nih.gov/snp) and the 1000 Genomes (http://www.ensembl.org/Homo_sapiens/) databases were surveyed.

### Expression analysis

To examine the expression pattern of the *MYH7B* and *ITGA7* genes, real-time PCR was performed on total RNAs from human adult tissues purchased from Stratagene and on total RNAs derived from the blood of family members using the LightCycler system DNA Engine Opticon 2 (MJ Research). The detailed protocol has already been published [21,22].

The expression levels were normalized to glyceraldehyde 3-phosphate dehydrogenase (*GAPDH*) to account for differences in the starting material and in the cDNA reaction efficiency. Agarose gel electrophoresis was performed to further confirm the specific PCR products.

The *MYH7B* and *ITGA7* transcript primers were the following:

MYH7B-cDNA-F 5′ GTCTGGGTGCCTGATGAACA 3′

MYH7B-cDNA-R 5′ CTCGTTCAGGTGCGTCATCA 3′

ITGA7-IS1-F 5′ GGATGGTGGGGAATGGAAGT 3′

ITGA7-IS1-R 5′ GGTCAGCAGGGTCCAAAGTT 3′

ITGA7-IS2-R 5′ GCGGGGGTCCTGCTCTTCT 3′

ITGA7-IS3-F 5′ CAGAGGCAGGCAGAAGGATT 3′

The primer ITGA7-IS2-R was combined with either ITGA7-IS1-F or ITGA7-IS3-F.

The GAPDH primers were as follows: forward primer, 5′ AGCCACATCGCTCAGACAC 3′, and reverse primer, 5′ GATCTCGCTCCTGGAAGATG 3′.

## Results

### Clinical features of patient V-4

The proband (V-4) is the first child of a family with a history of consanguinity (mother’s parents are first-degree cousins) and diabetes in the maternal line (grandmother, one great-uncle, one uncle and a first-degree cousin) without neuromuscular diseases (Figure 1). Since birth, she manifested hypotonia, poor sucking and persistent crying. Persistent arterial duct and patent foramen ovale were diagnosed at birth but resolved spontaneously, as shown by echocardiography at the age of 1 month. At 3 months of age, she was diagnosed with congenital dislocation of the left hip, and at 17 months of age, she was hospitalized because of body weight below the third percentile and hypoglycemia. The failure to thrive was due to difficulties in chewing and swallowing. ECG showed a long QTc (478 msec). Echocardiography showed a slight left ventricular (LV) dilatation, a moderate reduction of the LV global function (ejection fraction 40%), and a non-compacted aspect of the overall infero-lateral wall. She was administered ace-inhibitors, diuretics and digitalis, with a significant improvement in the clinical state. The karyotype and FISH were normal. The serum creatine kinase (CK) was within the normal range. The patient was followed up for 8 years**.**

At 8 years of age, she was admitted at our department for a neuromuscular and cardiological evaluation.

**Figure 2:**
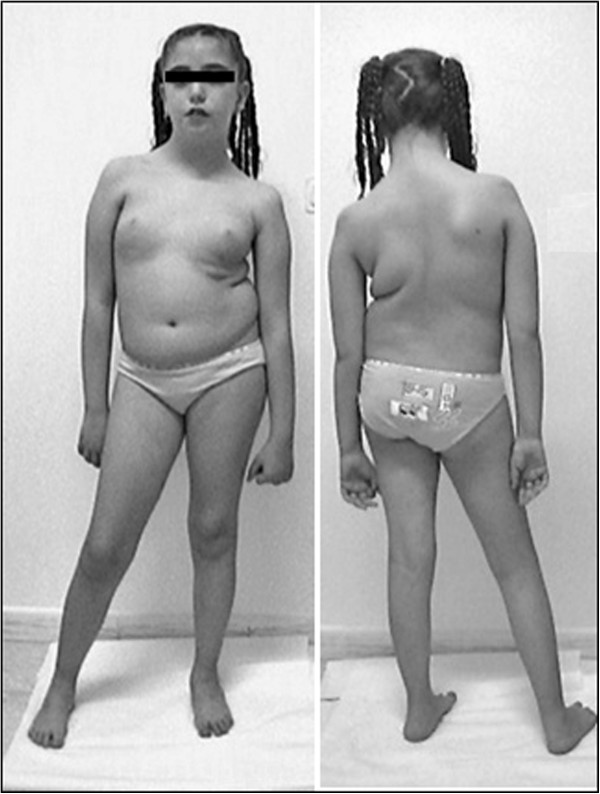
**The proband at 10 years of age.** Note the marked scoliosis, the leg length discrepancy and the left quadriceps hypotrophy; the abduction and elevation of the arms are impaired.

**Figure 3:**
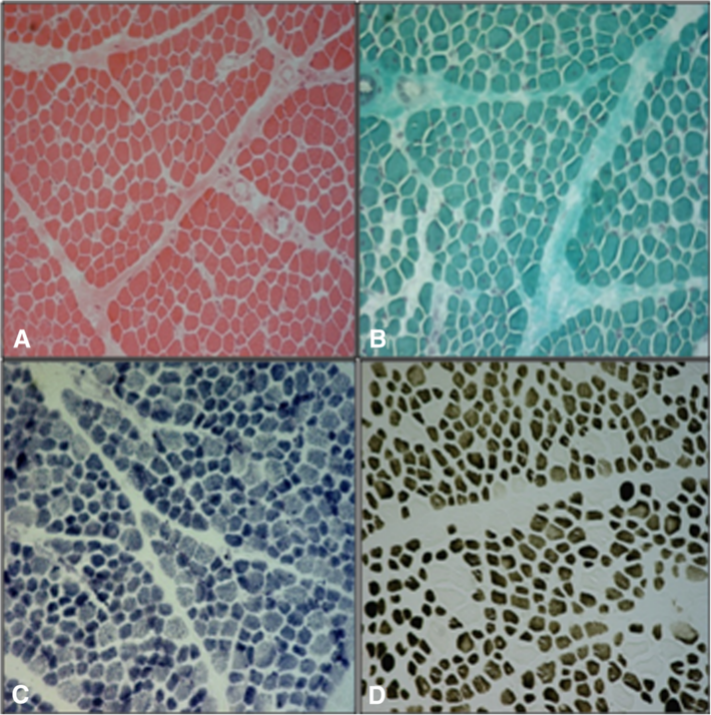
**Right quadriceps muscle biopsy (6-μm-thick cryostatic sections, 10×).** (**A**) H-E: moderate fiber caliber variability and diffuse mild fibrosis; (**B**) Trichrome of Gomori: absence of mitochondrial accumulation; (**C**) NADH-Tr: no oxidative enzymes change; (**D**) ATPase pH 4.3: a clear predominance of type I (dark) fibers, which show diameters smaller than those of type 2 fibers (clear).

The *neurologic examination* revealed the following: a waddling gait, an inability to stand up from a sitting position, a slight degree of weakness of the facial and masticator muscles, a mild weakness of the glutei and ileo-psoas and marked bilateral quadriceps. She developed marked scoliosis due to congenital hip dysplasia (Figure 2). Tendon reflexes were absent. Joints contractures had never been observed. A blood test confirmed hypoglycemia and normal levels of serum creatine kinase and isoenzymes. An EEG and a brain MRI **s**howed no abnormality. A quadriceps muscle biopsy displayed a predominance of type 1 fibers (76%), the mean diameter of which was 30% smaller than that of the type 2 fibers; the latter were hypertrophic for the age (mean diameter of 35 μm) (Figure 3). No internal nuclei, nemaline bodies or cores were observed. Dystrophin, emerin, calpain and lamin A/C were examined and found to be normal. This allowed us to exclude other neuromuscular disorders associated with the myopathy. These findings were compatible with the diagnosis of CFTD.

**Figure 4:**
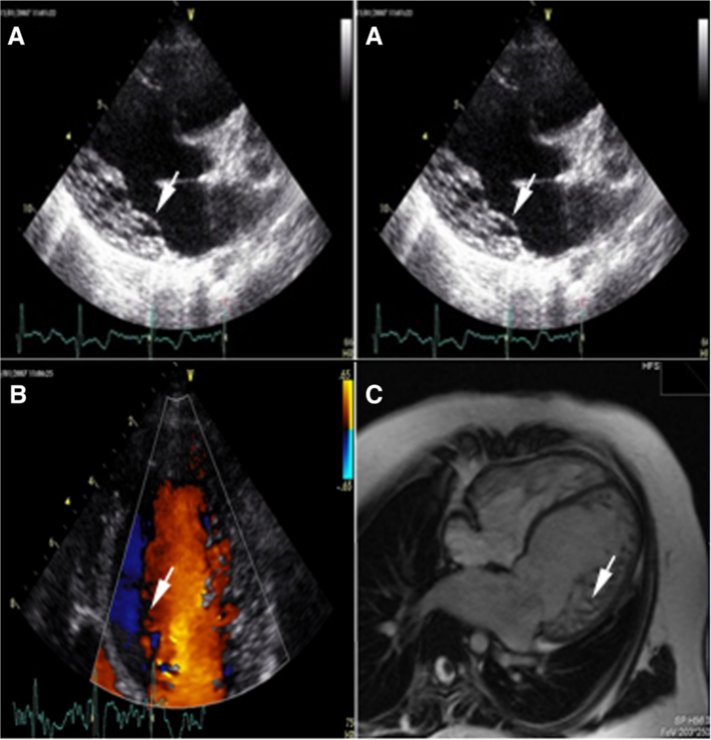
**Cardiological features.** (**A**) B-mode Echocardiography: ipertrabeculation/non-compaction left ventricle cardiomyopathy (white arrows). (**B**) Colordoppler-Echocardiography: blood flowing into the intertrabecular spaces (white arrow) communicating with the left ventricle lumen. (**C**) Cardiac MRI: a T1 weighted image clearly showing the left ventricle wall trabeculation (white arrow).

The *cardiological investigation* confirmed a long QTc and showed signs of left ventricular hypertrophy and repolarization abnormalities in the ECG. The echocardiography showed a dilated left ventricle (left ventricular end diastolic diameter z-score +3) with hypertrabeculation of the antero-lateral, postero-lateral and inferior walls (non-compacted/compacted ratio ~3), a severe impairment of the LV global function (ejection fraction 35%), a moderate mitral regurgitation, an abnormal mitral inflow pattern (E/A ratio 0.8), and a mild increase in the pulmonary artery pressure (40 mmHg) with normal non-invasive pulmonary wedge pressure (E/Ea ratio 8) (Figures 4A and 4B). A 24-hour Holter ECG monitoring revealed 5 short runs of supraventricular tachycardias. The patient was administered with carvedilol and ace-inhibitors, with the prescription of routine cardiac evaluations (every 4–6 months).

At the last cardiological evaluation (10 years of age), she was asymptomatic for dyspnoea (NYHA class I) and in therapy with carvedilol 6.25 mg ½ cp 2 times/day, losartan 50 mg ½ cp/day, spironolattone 25 mg ½ cp/day, furosemide 25 mg 1 cp/day and cardioaspirin 100 mg 1cp/day. The ECG confirmed a prolonged QTc (485 msec), and the echocardiography showed a non-compacted LV with 38% ejection fraction and slightly elevated pulmonary artery pressure (45 mmHg). NT-proBNP was elevated (1858 pg/ml). A cardiac MRI analysis confirmed the diagnosis of LVNC, with a significant reduction of ejection fraction to 40% (Figure 4C).

One month before the cardiovascular appointment, the patient (11 years old) died suddenly early in the morning, while lying in bed.

### Gene identification strategy

Whole-exome sequencing of the proband, affected by CFTD and LVNC, and her cousin, affected by LVNC, was used as a strategy to identify the responsible gene/s.

At first, we performed a thorough survey of all previously identified CFTD and LVNC genes to definitively exclude their involvement in the disease phenotype. Only intronic polymorphic variants were detected in *TPM3*, *ACTA1*, *TPM2, MYL2*, *MYL3*, *G4.5, DTNA*, *ZASP, ACTC1*, *MYBPC3* and *TMP1*. Both intronic and coding polymorphic variants were detected in *SEPN1*, *RYR1*, *MYH7*, *LMNA/C*, *TNNT2* and *TNNI3* (Table 1). After filtering the data with dbSNP130 and data from six in-house exomes (1 healthy individual and 5 individuals with unrelated diseases; Table 2), we performed two models of analysis to follow both phenotypes: LVNC that segregated as dominant and CFTD that showed a recessive mode of inheritance. For the dominant model, we matched data from both individuals (V-1/V-4) to obtain shared variants. In total, 93 autosomal non-synonymous coding variants were shared between the two samples. Only 56 of these variants were confirmed by direct sequencing in the two patients. Ten variants showed complete segregation with the LVNC phenotype in the family but were also found in the healthy population with a minor allele frequency (MAF) >1%. Only one of these, R890C in myosin, heavy chain 7B, cardiac muscle, beta (*MYH7B*), was found in the 1000 Genomes database with a MAF < 0.05% and was predicted to be a deleterious change by bioinformatics tools.

**Table 1:** Summary of the coding polymorphic variants found in CFTD-LVNC genes

**Gene**	**Amino acid change**	**rs**	**MAF**
SEPN1	C142Y	rs7349185	16%
SEPN1	P391P	rs760597	20%
SEPN1	N502K	rs2294228	28%
RYR1	L198L	rs2229139	39%
RYR1	P762P	rs3745847	38%
RYR1	T981T	rs2228069	40%
RYR1	N993N	rs2228070	24%
MYH7	I989I	rs7157716	35%
MYH7	K365K	rs735711	9%
MYH7	F244F	rs2069542	22%
LMNA	H454H	rs4641	20%
TNNT2	I101I	rs3729547	32%
TNNI3	E179E	rs3729841	4%

*MAF* minor allele frequency.

**Table 2:** Summary of the whole exome sequencing of CFTD-LVNC samples

**Data**	**V-1**	**V-4**	**V-1 + V-4**
Mapped Reads	52,921,718 (89%)	42,081,251 (90%)	
SNV	207.784	160,388	
dbSNPs	140.798	110,677	
coding variants	25.387	24,669	
Non synonym. (NS)	13.480	13,089	
Frame-Shift	196	182	
NS after filter dbSNPs	3923	3784	
NS after filter 6 exomes*	2983	2806	
Dominant Model			93
Dominant Model + validation**			56
Dominant Model (deleterious)			**1**
Recessive Model homozygous		46	
Recessive Model + validation		10	
Recessive Model (deleterious)		**1**	
Recessive Model heterozygous		50	
Recessive Model + validation		5	
Recessive Model (deleterious)		0	

*SNV* single nucleotide variation; *: non synonymous variants obtained after to filter with six in house genome; Dominant Model: number of gene in which at least one variant is shared between the two samples; Dominant Model + validation**: autosomal variants shared between the two samples and remaining after filtering with 1000 genome database and validated with direct sequencing; Dominant Model (deleterious): variants predicted as deleterious effect of the change with SIFT; Recessive Model: genes in which two alleles are mutated.

For the recessive model, only the proband was considered to be affected. We then selected all genes carrying two mutations. In total, 46 homozygous changes were selected, but only 10 were confirmed by direct sequencing and analyzed in the family samples. It is important to note that the parents of individual IV-14 (mother of the proband) are first-degree cousins, so she is homozygous for many variants and represents a good sample to filter data derived from the proband. In fact, 9 of the 10 variants were excluded because they were homozygous both in the proband and in her mother, who did not show the CFTD phenotype. Only the E882K variant, in the integrin, alpha 7 (*ITGA7*) gene, segregated with the CFTD phenotype in the family.

The compound heterozygous model produced 50 genes, of which only 5 continued to carry two mutations after direct sequencing analysis; none of the 50 genes segregated with the disease phenotype in the family because they were also present in the sister of the proband (V5), who showed no CFTD phenotype.

### The myosin heavy chain 7B (*MYH7B*) gene

*MYH7B* [GenBank: NG_016984.1] is located on chromosome 20 and is split into 45 exons, 43 of which code for protein. The nucleotide substitution c.2668 C > T is located in exon 27 and causes the non-synonymous change of the amino acid arginine at position 890 into cysteine (R890C).

**Figure 5:**
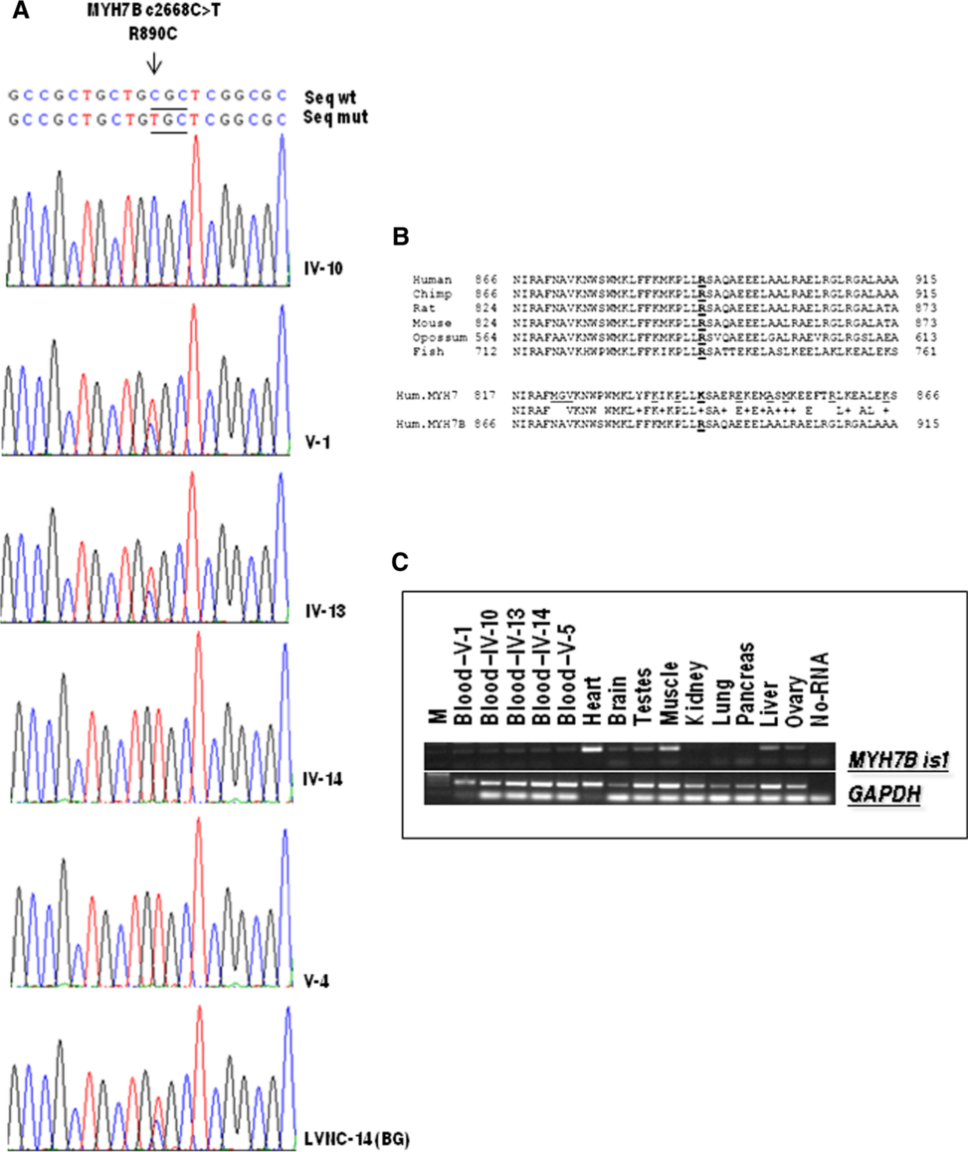
**Molecular analysis of MYH7B gene mutation.** (**A**) Mutation analysis of the *MYH7B* gene. Arrowheads mark the sites of base alterations. The DNA sequences of the family subjects are shown; the pedigree number is indicated on the right. (**B**) Evolutionary conservation analysis. Arginine 890 is underlined. The *MYH7* amino acids mutated in cardiomyopathy are shown in red. (**C**) *MYH7B* mRNA expression in human tissues and in RNA derived from family members. M indicates the 1 kb Plus marker (Fermentas).

The inheritance of the R890C mutation was examined by Sanger sequencing of the DNA of the family individuals (Figure 5A). It was found to be homozygous in samples IV-14 and V-4 and to be heterozygous in IV-13, V-1 and V-5; it was absent in IV-10. This finding suggests the segregation of the mutation with the LVNC disease phenotype in our family; however, its reduced penetrance and variable expressivity need to be considered because the mutation is also carried by the asymptomatic subject IV-13. To further validate our finding, we analyzed the R890C mutation in a panel of 24 unrelated LVNC patients from the same geographical area and found it in patient LVNC-14 (BG), who showed a classical LVNC phenotype with severe systolic dysfunction (25%) (Figure 5A).

We then analyzed 600 chromosomes from an unrelated healthy population from the same geographical origin. This mutation was absent in this panel but was present in the 1000 Genomes database with a MAF < 0.006%.

Most variants underlying rare Mendelian diseases either affect highly conserved sequences and/or are predicted to be deleterious. For this reason, we analyzed the R890C mutation with the SIFT and PolyPhen2 software to confirm a deleterious effect. At this position (R890), only arginine (R), lysine (K), serine (S), asparagine (N) or glutamine (Q) are tolerated (data not shown). The PolyPhen-2 analysis supported the notion that this variant is “damaging”, with the highest probability score of 1.

**Figure 6:**
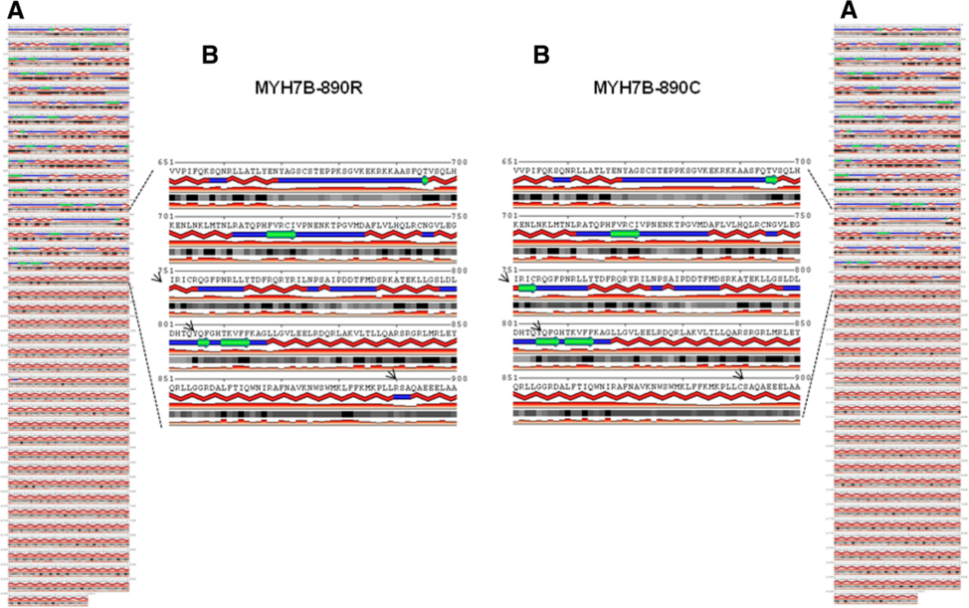
**Secondary structures prediction of MYH7B.** (**A**) Secondary structures predictions for the MYH7B-890R (left) and MYH7B-890C (right) proteins. The program also performed an accurate prediction of the real-valued relative solvent accessibility. The 890 R to C amino acid substitution changes the beta strands (green arrows) and alpha helix (red lines) structures of the MYH7B protein. All changes are indicated with black arrows. **(B)** Enlarged area with the region containing the mutation highlighted.

The *MYH7B* gene belongs to the MYH gene family, which, in humans, also includes the *MYH6* and *MYH7* genes, both clustered on chromosome 14. Evolutionary conservation analysis shows high similarity for these proteins. Interestingly, the amino acid arginine (R) at position 890 is highly conserved in all species, but in *MYH6* and *MYH7* and their homologs, the arginine (R) is replaced with lysine (K) (Figure 5B). However, lysine is one of the amino acids that SIFT predicted to be tolerated at this protein position. Using a bioinformatics simulation model (http://minnou.cchmc.org/), we demonstrated that the R890C mutation induces a conformational change in the MYH7B molecule [GenPept: AAI51243.1], leading to the alteration of the beta sheet and alpha helix structures (Figure 6).

A significant expression of the *MYH7B* gene was observed in heart and muscle tissues, and very low expression was observed in the brain, testes, ovary, liver and blood. No expression was observed in the kidney, lung and pancreas. No difference in expression was observed in blood RNA derived from both affected and healthy subjects from our family (Figure 5C).

### The integrin, alpha 7 (*ITGA7*) gene

The *ITGA7* gene [GeneBank: NG_012343.1] is located on chromosome 12 and is split into 28 exons, 26 of which code for protein. This gene has 23 transcripts, but only 10 are protein coding. The homozygous nucleotide substitution c.2644 G > A is located in exon 20 and causes the non-synonymous change E882K in transcript 1 [GeneBank: NM_001144996.1]. The position of this mutation is E886K in isoform 2 [GeneBank: NM_002206.2] and E789K in isoform 3 [GeneBank: NM_001144997.1].

**Figure 7:**
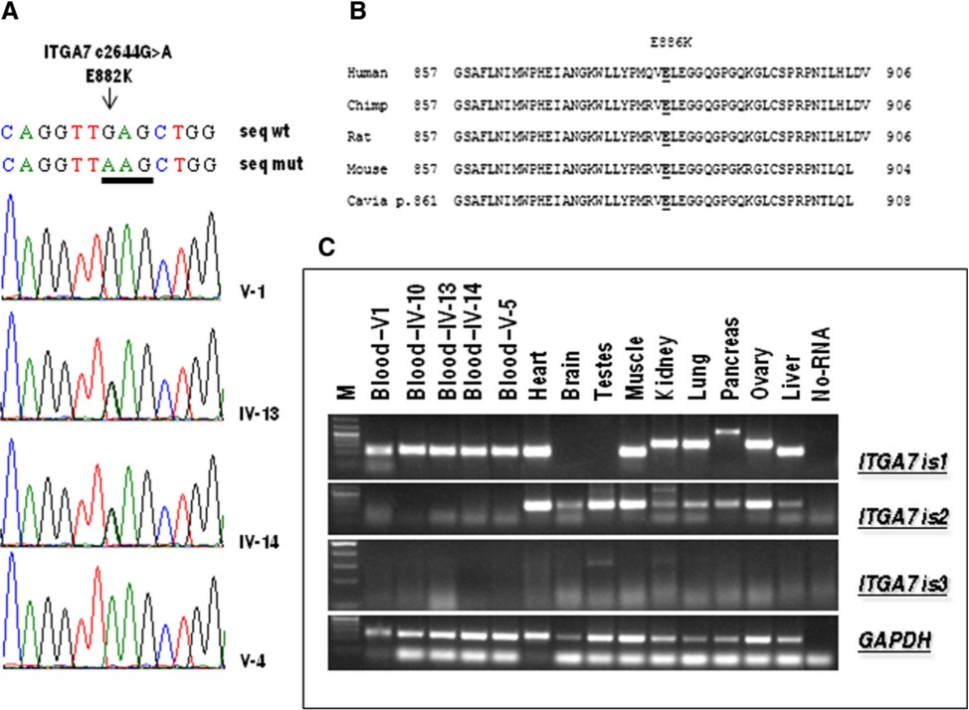
**Molecular analysis of ITGA7 gene mutation.** (**A**) Mutation analysis of the *ITGA7* gene. Arrowheads mark the sites of base alterations. The DNA sequences of the family subjects are shown; the pedigree number is indicated on the right. (**B**) Evolutionary conservation analysis. The glutamic acid at position 882 is underlined. (**C**) *ITGA7* mRNA expression in human tissues and in RNA derived from family members. M indicates the 1 kb Plus marker (Fermentas).

The inheritance of this mutation was examined by Sanger sequencing of the DNA of the family individuals. Our analysis confirmed that this mutation was present in a homozygous form only in the proband with the CFTD phenotype; her parents were heterozygous for this change (Figure 7A).

**Figure 8:**
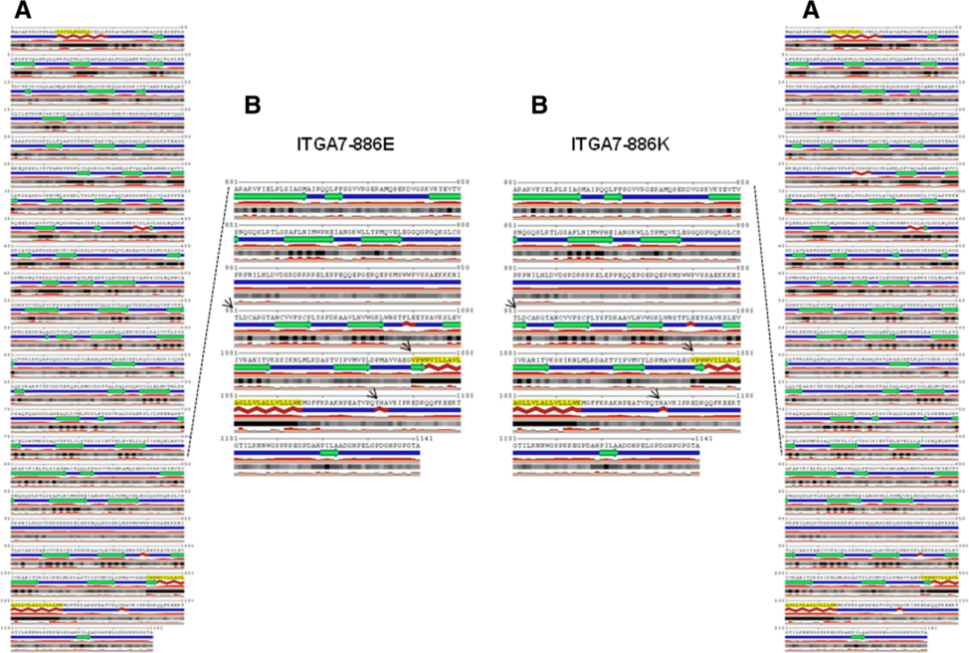
**Secondary structures prediction of ITGA7.** (**A**) Secondary structures predictions for the ITGA7-882E (left) and ITGA7-882 K (right) proteins. The program also performed an accurate prediction of the real-valued relative solvent accessibility. The 882 E to K amino acid substitution changes the beta strands (green arrows) and alpha helix (red lines) structures of the ITGA7 protein. All changes are indicated with black arrows. (**B**) Enlarged area with the region containing the mutation highlighted.

In the 1000 Genomes database, this change is reported as number rs144983062; the heterozygous genotype C/T was found in 12 of the 4540 samples analyzed (freq of the CT genotype = 0.003), and the homozygous genotype TT was not found. We analyzed 600 chromosomes from an unrelated healthy population from the same geographical area of the proband but did not find the mutation. Therefore, we analyzed the E882K mutation with the SIFT and PolyPhen2 software, which confirmed a deleterious effect of this variant. Evolutionary conservation analysis showed that the missense change E882K affects a highly conserved residue of the integrin alpha 7 protein (Figure 7B). Moreover, bioinformatics simulation modeling demonstrated that the E882K mutation induces a conformational change in the ITGA7 molecule, resulting in the alteration of the beta sheet structures (Figure 8).

Expression profiling was performed for three transcripts of the gene. Isoform 1 was significantly expressed only in heart, muscle, liver and blood tissues. No difference in expression was detected between blood RNAs derived from affected and healthy subjects of our family. Isoform 2 was significantly expressed in heart, muscle, testes and ovary tissues; low levels of expression were detected in the brain, kidney, lung, pancreas and liver; no expression was detected in the blood. Isoform 3 was detected at very low levels of expression only in the testes and kidney (Figure 7C).

## Discussion

We describe an unusual association between CFTD and LVNC and link this unusual severe phenotype to digenic inheritance, a novel type of transmission that is emerging with the application of novel powerful genomic technologies, such as whole-exome sequencing [23]. The CFTD myopathy is a genetically heterogeneous disorder characterized by relative hypotrophy of type 1 muscle fibers compared to type 2 fibers in skeletal muscle biopsies [1]. The diagnosis of CFTD is made for exclusion because these findings are not specific and can be found in several neuromuscular diseases. The term “fiber size disproportion” has been suggested for describing this specific histological picture, reserving the term CFTD for those cases in which no secondary cause can be found [1]. Cardiovascular involvement has been rarely reported in patients with CFTD myopathy. Banwell et al. described 2 unrelated children with cardiac involvement [24]. One child, with a dilated type cardiomyopathy, developed an intractable congestive heart failure, necessitating cardiac transplantation at the age of 13 years. The second, a 1-year-old child without cardiomyopathy or congenital heart diseases, developed a high-rate atrial fibrillation, requiring treatment with digoxin. The association of DCM with CFTD has also been reported in two Japanese patients [25,26]. However, a defined geno-phenotype correlation has not been established in these cases. LVNC has been reported in patients with different types of NMDs, including dystrophinopathy, laminopathy, zaspopathy, myotonic dystrophy, Barth syndrome, Friedreich ataxia, Charcot-Marie-Tooth disease, and metabolic and mitochondrial disorders [27,28].

Nevertheless, considering the rarity of these pathological conditions, which are caused by a premature arrest of skeletal and cardiac muscle development, the hypothesis of a common pathogenesis is intriguing.

Our data suggest that rather than the action of a single gene, a synergic effect of homozygous mutations in two genes, i.e., myosin heavy chain 7B (*MYH7B*) and integrin alpha 7 (*ITGA7*), underlies the phenotype observed in the proband (V-4). Both genes are crucial for the physiological development of skeletal and cardiac muscles. However, a single-gene mutation responsible for both DCM/LVNC and CFTD in other patients cannot be conclusively ruled out.

Myosin, the molecular motor responsible for muscle contraction, exists in multiple forms, which dictate muscle properties, such as shortening velocity and contractile force. The majority of MYH genes known to be present in mammals are associated in two highly conserved gene clusters [29,30]. *MYH6* and *MYH7* are two tandemly arrayed genes located on human chromosome 14, which code for the cardiac myosins, α- and β-MYH; β-MYH is also expressed in slow skeletal muscles. Another gene cluster, located on human chromosome 17, codes for the six skeletal myosins, including the adult fast 2A-, 2X- and 2B-MYH, the developmental embryonic and neonatal/perinatal isoforms, and MYH13, an isoform expressed specifically in extraocular muscles. Three additional genes coding for sarcomeric MYHs, i.e., *MYH7B*, *MYH15* and *MYH16*, have been discovered recently. We showed that *MYH7B* is expressed in the adult human heart and muscle; moreover, Warkman and coworkers recently determined that *MYH7B* is expressed in the myocardium [31]. Developmental analysis showed Myh7b expression in cardiac and skeletal muscles of Xenopus, chick and mouse embryos and in smooth muscle tissues during the later stages of mouse embryogenesis [31]. Heterozygous mutations in eight sarcomere proteins (*MYH7, ACTC1, TNNT2, TNNI3, MYL2, MYL3, MYBPC3 and TPM1)* have been identified in a significant proportion of patients with LVNC in adults and children [14-16,32,33]. Approximately 20% of LVNC patients carried a mutation in *MYH7*, which was the most prevalent LVNC disease gene; missense mutations were the most prevalent types of mutations in all sarcomere proteins analyzed [16,32]. LVNC is characterized by a trabecular meshwork and deep intertrabecular myocardial recesses communicating with the left ventricular (LV) cavity [18]. Clinical features range from a non-penetrant disease in adult carriers to heart failure, arrhythmia and thromboembolism [34,35]. The penetrance of a mutation is defined as the percentage of mutation carriers expressing a phenotype, and most autosomal-dominant cardiomyopathies are characterized by incomplete penetrance or more age-related penetrance [36]. There is also variable expressivity in cardiomyopathies, and there can even be large differences among relatives of the same family (intrafamilial variability) who carry the same mutation. In our family, high phenotypic variability and reduced penetrance were observed.

This is the first indication that a mutation in the *MYH7B* gene causes LVNC cardiomyopathy. We demonstrated that the arginine at position 890 of the *MYH7B* gene is highly conserved in all species; this region is also conserved in the *MYH7* gene, which, when mutated, causes LVNC. These data further support the concept that sarcomere genes are associated with LVNC.

Integrin α7β1 is a specific cellular receptor for the basement membrane protein laminin-1 and for the laminin isoforms −2 and −4 [37,38]. The α7 subunit is expressed mainly in skeletal and cardiac muscles and has been suggested to be involved in differentiation and migration processes during myogenesis [39-41].

Mice homozygous for a null allele of the Itga7 gene are viable and fertile, indicating that the α7β1 integrin is not essential for myogenesis. However, a histological analysis of skeletal muscle revealed typical symptoms of a progressive muscular dystrophy starting soon after birth, but with a distinct variability in different muscle types [42]. The knock-down of zebrafish Itga7 results in muscle fiber detachments similar to those observed in lama2 and lama4-deficient embryos [43]. The human deficiency in integrin α7 causes a mild disorder that is best characterized as congenital myopathy. Three patients with mutations in the *ITGA7* gene have been described. One patient had splice mutations on both alleles: one mutation caused a 21-bp insertion in the conserved cysteine-rich region, and the other caused a 98-bp deletion. A second patient was a compound heterozygote for the same 98-bp deletion and had a 1-bp frame-shift deletion in the other allele. The third patient showed a marked deficiency in the *ITGA7* mRNA, but no mutations in the coding region were described. In muscle biopsies, patients 1 and 3 showed a poorly defined congenital myopathy, which was associated with mental retardation in patient 1. Patient 2 presented a clinical and pathological picture typical of muscular dystrophy, with substantial fatty acid replacement and fiber size variation (MIM 613204) [44].

Our proband (V-4) harbored a homozygous missense mutation in a highly conserved region of the protein. The typical pattern of CFTD was observed in the muscle biopsy, characterized by the predominance of type 1 fibers with smaller calibers than type 2 fibers, with no evidence of either congenital muscular dystrophy or muscular dystrophy. Clinically, she had no symptoms of a delay in mental development, which occurs only in a few cases of CFTD. Our proband and the above-mentioned cases, showing muscle disorders present from birth, all support the important role of *ITGA7* in myogenesis. The differences in their phenotypes may be related to their diverse patterns of gene mutation.

## Conclusions

This study identifies two novel disease genes. Mutation in *MYH7B* causes a classical LVNC phenotype, whereas mutation in *ITGA7* causes CFTD. The synergic effect of these two mutations causes the severe phenotype observed in the proband. This study provides new insights into the genetics of cardiomyopathy and congenital myopathy.

## Competing interests

All authors have non-financial interests that may be relevant to the submitted work.

## Authors’ contributions

TE, DF, FN and FG performed the sequencing and expression analyses. SS, GL, AV, DD, OF GP, and GDI recruited the family described herein and collected the clinical data. TE, SS, GL, FG and GDI oversaw all aspects of the research. TE and GDI initiated, planned and coordinated the study. TE, SS, GL and GDI wrote the manuscript. All authors read, edited and approved the final version of the manuscript.
